# Potential Mechanisms of Triptolide against Diabetic Cardiomyopathy Based on Network Pharmacology Analysis and Molecular Docking

**DOI:** 10.1155/2021/9944589

**Published:** 2021-12-07

**Authors:** Ning Zhu, Bingwu Huang, Liuyan Zhu, Yi Wang

**Affiliations:** ^1^Department of Cardiology, The Third Affiliated Hospital of Shanghai University (Wenzhou People's Hospital), No. 299 Guan Road, Wenzhou, 325000 Zhejiang Province, China; ^2^Department of Anesthesiology and Perioperative Medicine, The Second Affiliated Hospital and Yuying Children's Hospital of Wenzhou Medical University, 109 Xueyuan West Road, Wenzhou, 325000 Zhejiang Province, China; ^3^Department of General Practice, The Third Affiliated Hospital of Shanghai University (Wenzhou People's Hospital), No. 299 Guan Road, Wenzhou, 325000 Zhejiang Province, China

## Abstract

The incidence of heart failure was significantly increased in patients with diabetic cardiomyopathy (DCM). The therapeutic effect of triptolide on DCM has been reported, but the underlying mechanisms remain to be elucidated. This study is aimed at investigating the potential targets of triptolide as a therapeutic strategy for DCM using a network pharmacology approach. Triptolide and its targets were identified by the Traditional Chinese Medicine Systems Pharmacology database. DCM-associated protein targets were identified using the comparative toxicogenomics database and the GeneCards database. The networks of triptolide-target genes and DCM-associated target genes were created by Cytoscape. The common targets and enriched pathways were identified by the Gene Ontology (GO) and Kyoto Encyclopedia of Genes and Genomes (KEGG) pathway enrichment analyses. The gene-gene interaction network was analyzed by the GeneMANIA database. The drug-target-pathway network was constructed by Cytoscape. Six candidate protein targets were identified in both triptolide target network and DCM-associated network: STAT3, VEGFA, FOS, TNF, TP53, and TGFB1. The gene-gene interaction based on the targets of triptolide in DCM revealed the interaction of these targets. Additionally, five key targets that were linked to more than three genes were determined as crucial genes. The GO analysis identified 10 biological processes, 2 cellular components, and 10 molecular functions. The KEGG analysis identified 10 signaling pathways. The docking analysis showed that triptolide fits in the binding pockets of all six candidate targets. In conclusion, the present study explored the potential targets and signaling pathways of triptolide as a treatment for DCM. These results illustrate the mechanism of action of triptolide as an anti-DCM agent and contribute to a better understanding of triptolide as a transcriptional regulator of cytokine mRNA expression.

## 1. Introduction

Diabetic cardiomyopathy (DCM) is defined as abnormal cardiac structure and function in the absence of other cardiac risk factors [1]. Although DCM has been well studied in the past decades, it remains a significant cause of morbidity and mortality in patients with diabetes [2]. A growing number of studies have shown that diverse mechanisms are involved in diabetes-associated cardiac dysfunction, including systemic insulin resistance, oxidative stress, inflammation, activation of the renin angiotensin aldosterone system, and dysregulation of the immune system [3]. At present, the management of high blood glucose levels in patients with DCM is tailored to minimize the risk of cardiovascular complications [4]. Western drugs can reduce the risk of cardiac complications without the blood glucose-lowering properties. It has been reported that inflammation plays a key role in DCM. Thus, the inhibition of inflammation may be a promising therapeutic strategy for DCM.

Traditional Chinese medicine (TCM), characterized by its multitarget nature, has been used to treat different types of diseases in China for thousands of years [5, 6]. Recent evidence has suggested that TCM is effective in treating cardiovascular diseases, such as DCM [7, 8]. Triptolide is a diterpenetriepoxide extracted from the herb *Tripterygium wilfordii* Hook. F [9]. The therapeutic effect of triptolide on DCM and the targets and pathways related to the anti-DCM effect has not been well identified [10, 11].

**Figure 1:**
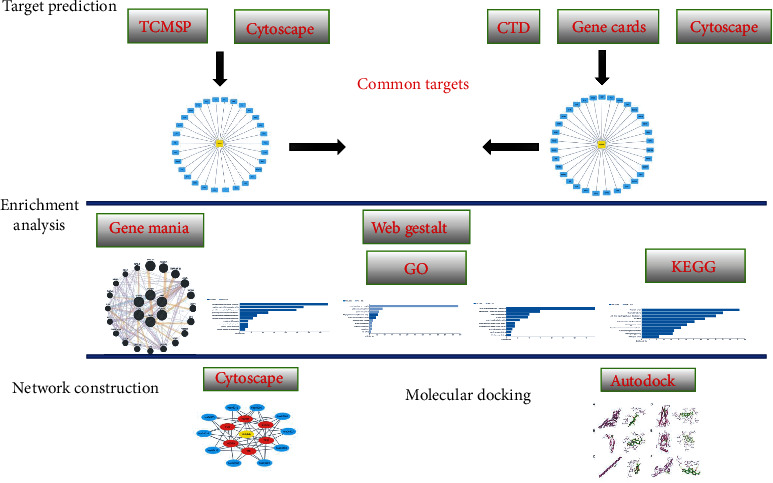
The procedures of the systematic strategies to elucidate the mechanisms of triptolide in the treatment of DCM. Targets related to triptolide and DCM were collected from online databases. The common targets of triptolide treating DCM were identified. Then, KEGG pathway enrichments were performed, and a network of triptolide-targets-KEGG was constructed. Finally, molecular dockings between triptolide and candidate target proteins (STAT3, VEGFA, FOS, TNF, TP53, and TGFB1) were conducted.

Network pharmacology-based drug discovery is a novel, promising, cost-effective approach for drug development. In contrast to the “one target, one drug” principle, network pharmacology uses the “network target, multicomponent” mode by combining systems biology, bioinformatics, and pharmacology. Network pharmacology has been widely used to explore the mechanism of the protective action of TCM on diseases. In the present study, the candidate targets of triptolide in the treatment of DCM were predicted by network pharmacology. The pathway enrichment analysis was performed to reveal potential therapeutic strategies for DCM. The procedures of this study are shown in Figure 1.

## 2. Material and Methods

### 2.1. The Chemical Structure of Triptolide

**Figure 2:**
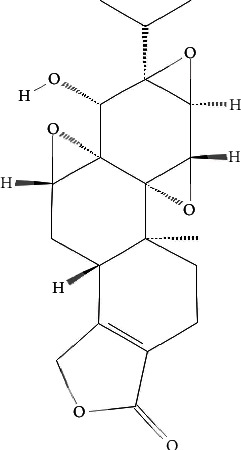
The chemical structure of triptolide. The PubChem database (http://pubchem.ncbi.nlm.nih.gov) was used to retrieve the 2D chemical structure of triptolide.

PubChem (https://pubchem.ncbi.nlm.nih.gov) is an open chemical database that provides information on compound structures and descriptive data. The PubChem database was used to retrieve the 2D chemical structure of triptolide (Figure 2).

### 2.2. The Targets of Triptolide

The Traditional Chinese Medicine Systems Pharmacology (TCMSP, http://lsp.nwu.edu.cn/tcmsp.php) database is a systems pharmacology platform of TCMs and related compounds [12] that summarizes the absorption, distribution, metabolism, and excretion (ADME) properties of drugs with potential biological effects. The oral bioavailability (OB), intestinal epithelial permeability (Caco-2 cells), drug likeness (DL), and blood-brain barrier (BBB) permeability of triptolide were obtained from TCMSP. The targets of triptolide were also collected from TCMSP.

### 2.3. The Identification of Therapeutic Targets of DCM

The therapeutic targets of DCM were identified using the comparative toxicogenomics database (CTD; http://ctdbase.org/) [13] and the GeneCards database (http://www.genecards.org/) [14] with a rank score of ≥30. The targets were filtered to retain only Homo sapiens genes.

### 2.4. GeneMANIA Analysis

GeneMANIA is a website for generating hypotheses based on gene function, analyzing gene lists, and prioritizing genes for functional assays [15]. In this study, GeneMANIA was used to construct a gene-gene interaction network to evaluate their functions. The organism was set to Homo sapiens, and potential candidate genes were entered in the search bar of GeneMANIA. The output of the network was then downloaded.

### 2.5. Gene Function and Pathway Enrichment Analyses

The WEB-based Gene Set Analysis Toolkit (WebGestalt, http://www.webgestalt.org/option.php) is a powerful tool for functional enrichment analysis, supporting 12 organisms, 324 gene identifiers from various databases and technology platforms, and 150,937 functional categories from public databases and computational analyses [16]. Potential candidate targets were uploaded to the WebGestalt server for the Gene Ontology (GO) and Kyoto Encyclopedia of Genes and Genomes (KEGG) analyses. The method of Over Representation Analysis (ORA) was adopted. A false discovery rate-adjusted *P* value of less than 0.05 indicates statistical significance.

### 2.6. Network Construction

Cytoscape (version 3.7.1) was used to construct a triptolide-target-pathway network and to analyze the relationships among compounds, targets, and diseases [17].

### 2.7. Molecular Docking

The crystal structures of candidate proteins were downloaded from the RCSB Protein Data Bank (http://www.pdb.org/) and modified using Autodock Tools (version 1.5.6) to add hydrogen, calculate Gasteiger electric charges, and combine nonpolar hydrogen. Autodock Tools was also used to add hydrogen and electric charges to triptolide. Autodockvina (version 1.1.2) was used to perform docking between triptolide and candidate target proteins.

## 3. Results

### 3.1. The Targets of Triptolide and Network Construction

**Figure 3:**
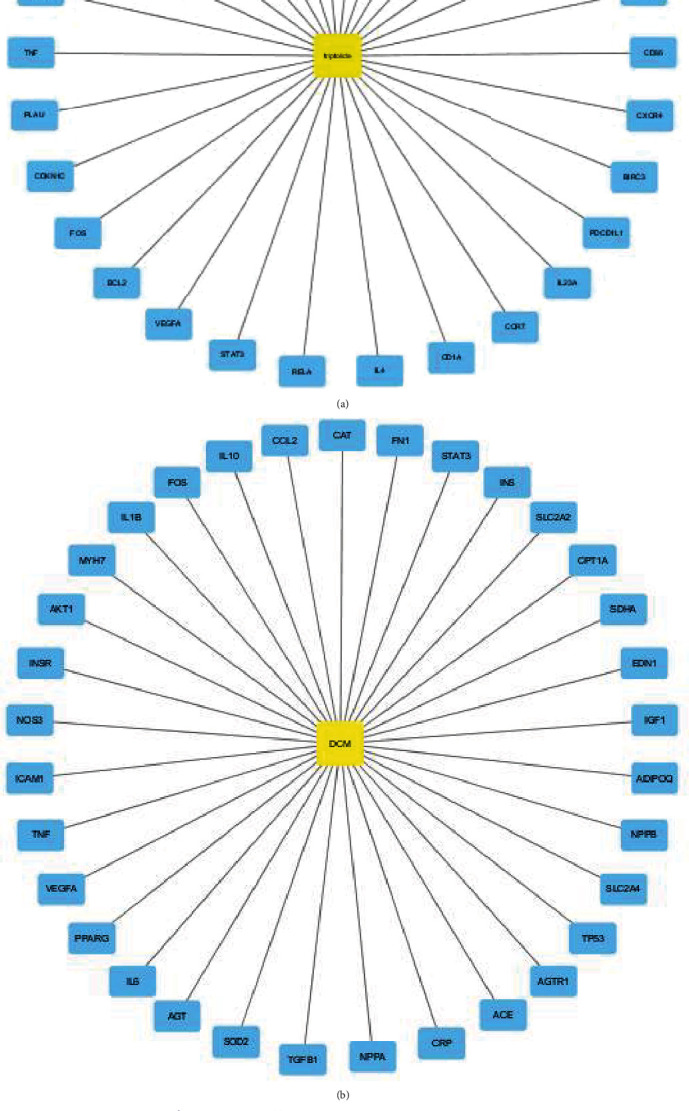
The prediction of common targets of triptolide acting on DCM. (a) Triptolide target network was constructed by Cytoscape. (b) DCM target network was constructed by Cytoscape. (c) Venny diagram of DCM targets collected from CTD and GeneCards database. (d) Venny diagram of triptolide and DCM intersection targets.

The chemical structure of triptolide was downloaded from the PubChem database (CID: 107985). A total of 30 targets of triptolide were predicted using the TCMSP database (Supplemental Table 1). The ADME properties of triptolide were as follows: molecular weight (MW), 360.44; AlogP, 0.87; Hdon, 1; Hacc, 6; OB (%), 51.29; Caco-2, 0.25; BBB, -0.19; DL, 0.86; FASA, 0.28; TPSA, 84.12; RBN, 1; HL, 4.14 (Table 1). These targets were imported into Cytoscape to plot the triptolide target network diagram (Figure 3(a)).

### 3.2. The Therapeutic Targets of DCM

A total of 153 and 138 targets were identified to be related to DCM in the CTD and GeneCards database, respectively, and the common targets were collected (Supplemental Table 2). The Venn diagram of the intersection of DCM-associated targets showed that there were 33 coincidence targets (Figure 3(b)). They were imported into Cytoscape to plot the DCM-associated target network diagram (Figure 3(c)).

### 3.3. Targets Related to the Treatment of DCM by Triptolide

The Venn diagram of the intersection of DCM-associated targets and the triptolide target network showed that there were six common candidate targets (STAT3, VEGFA, FOS, TNF, TP53, and TGFB1) (Figure 3(d), Table 2).

### 3.4. GeneMANIA Analysis

**Figure 4:**
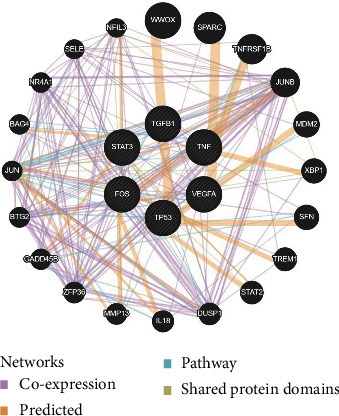
GeneMANIA analysis of gene–gene interaction network for candidate targets. Black nodes indicated target proteins and connecting colors suggested different correlations. Genes in black circles were query terms while these in gray circle represented genes associated with query genes. The purple lines represent coexpression. The golden lines represent predicted. The blue lines represent pathway. The green lines represent shared protein domains.

A gene-gene interaction network of the six common candidate targets was constructed, and their functions were analyzed using the GeneMANIA database (Figure 4). The 12 central nodes representing common targets were surrounded by 20 nodes representing genes that greatly correlated with the targets in terms of coexpression, prediction, pathway, and shared protein domains. The top five genes displaying the greatest correlations with these targets were WW domain-containing oxidoreductase (*WWOX*), secreted protein acidic and rich in cysteine (*SPARC*), TNF receptor superfamily member 1B (*TNFRSF1B*), receptor for activated C kinase 1 (*JUNB*), and mouse double minute 2 (*MDM2*).

### 3.5. GO and KEGG Analyses

**Figure 5:**
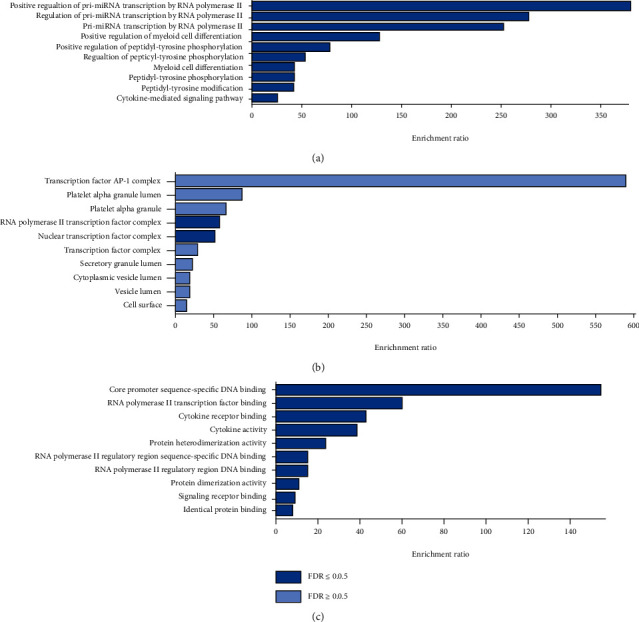
GO analysis of candidate target genes. (a) Biological process categories. (b) Cellular component categories. (c) Molecular function categories. The bar chart plots the enrichment results vertically with the bar width equal to enrichment ratio in ORA. FDR-adjusted *P* value < 0.05 indicates the enrichment degree has statistically significance.

**Figure 6:**
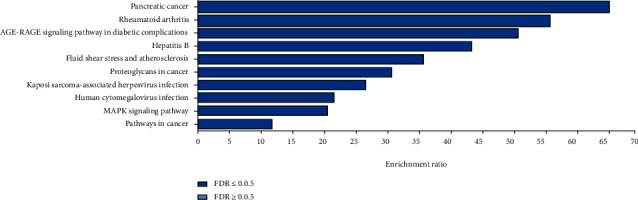
KEGG pathway analysis of candidate target genes. The bar chart plots the enrichment results vertically with the bar width equal to enrichment ratio in ORA. FDR-adjusted *P* value < 0.05 indicates the enrichment degree had statistically significance.

The targets of triptolide related to DCM were imported into the WebGestalt database for GO analysis. Ten biological processes were identified, including positive regulation of pri-miRNA transcription by RNA polymerase II, regulation of pri-miRNA transcription by RNA polymerase II, pri-miRNA transcription by RNA polymerase II, positive regulation of myeloid cell differentiation, positive regulation of peptidyl-tyrosine phosphorylation, regulation of peptidyl-tyrosine phosphorylation, myeloid cell differentiation, peptidyl-tyrosine phosphorylation, peptidyl-tyrosine modification, and cytokine-mediated signaling pathway (Figure 5(a)). In addition, two cellular components were identified, including RNA polymerase II transcription factor complex and nuclear transcription factor complex (Figure 5(b)). Ten molecular functions were also identified, including core promoter sequence-specific DNA binding, RNA polymerase II transcription factor binding, cytokine receptor binding, cytokine activity, protein heterodimerization activity, RNA polymerase II regulatory region sequence-specific DNA binding, RNA polymerase II regulatory region DNA binding, protein dimerization activity, signaling receptor binding, and identical protein binding (Figure 5(c)). The KEGG analysis showed that 10 pathways were enriched, including pancreatic cancer, rheumatoid arthritis, AGE-RAGE signaling pathway in diabetic complications, hepatitis B, fluid shear stress and atherosclerosis, proteoglycans in cancer, Kaposi sarcoma-associated herpesvirus infection, human cytomegalovirus infection, MAPK signaling pathway, and pathways in cancer (Figure 6).

### 3.6. Network Construction

**Figure 7:**
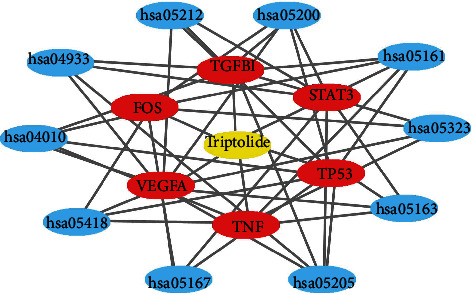
The construction of triptolide-target-pathway network. The yellow rectangles represent triptolide. The red rectangles represent three core targets. The blue rectangles show the top 10 KEGG pathways of triptolide treating DCM. The gray lines represent their interaction.

A triptolide-target-pathway network was constructed by Cytoscape based on the targets and the results of pathway enrichment analyses. As shown in Figure 7, the triptolide-target-pathway interaction network has 17 nodes and 50 edges. The yellow, red, and blue circles represent triptolide, target genes, and pathways, respectively.

### 3.7. Molecular Docking

**Figure 8:**
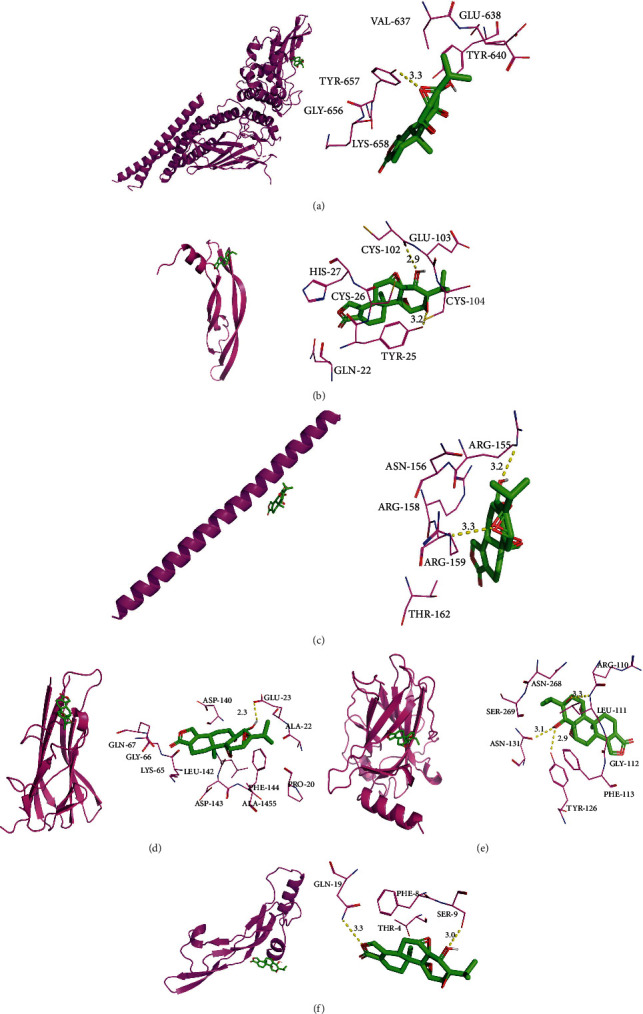
Molecular models of triptolide binding to the predicted targets (a) STAT3 (PDB: 6NJS), (b) (PDB:3V2A), (c) FOS (PDB:1A02), (d) TNF (PDB:5M2J), (e) TP53 (PDB: 6RZ3), and (f) TGF*β*1 (PDB: 4KV5). The yellow dashed lines show H-bonds, and the red dashed lines show *π*-*π* interactions, with interaction distances indicated above the lines.

The crystal structures of potential targets, including STAT3 (PDB: 6NJS), VEGFA (PDB: 3V2A), FOS (PDB: 1A02), TNF (PDB: 5M2J), TP53 (PDB: 6RZ3), and TGF*β*1 (PDB: 4KV5), were collected from the RCSB Protein Data Bank (Figure 8). Triptolide binds to STAT3 with a binding pocket consisting of TYR (3.3 Å). Triptolide binds to VEGFA with a binding pocket consisting of CYS-120 (2.9 Å) and CYS-104 (3.2 Å). Triptolide binds to FOS with a binding pocket consisting of ARG-155 (3.2 Å) and ARG-155 (3.3 Å). Triptolide binds to TNF with a binding pocket consisting of GLU-23 (2.3 Å). Triptolide binds to TP53 with a binding pocket consisting of ARG-110 (3.3 Å), ASN-131 (3.1 Å), and TYR-126 (2.9 Å). Triptolide binds to TGF*β*1 with a binding pocket consisting of GLN-19 (3.3 Å) and SER-9 (3.0 Å).

## 4. Discussion

Cardiovascular complications are the leading cause of mortality and morbidity in patients with diabetes. While ischemia dominates the cardiac complications of diabetes, it is well accepted that the risk for developing heart failure is also increased in the absence of overt myocardial ischemia and hypertension or is accelerated in the presence of these comorbidities [18]. Many potential mechanisms have been proposed for the pathogenesis of DCM, and some interventions have been developed to treat DCM in preclinical models. However, the “one target, one drug” approach is not effective due to the complex etiology and pathogenic mechanisms of DCM [19]. Network pharmacology as an emerging approach for drugs research via big data analysis contributes to a better understanding of the pharmacological mechanisms of drugs [20]. Hence, it has been widely applied in TCM research. TCM has been reported to have therapeutic effects for DCM [21–23]. Although the protective role of triptolide in DCM has been identified, the pharmacological mechanisms underlying the beneficial effects of triptolide on DCM are still unknown [10, 11]. In this study, we constructed a triptolide-DCM target network and performed pathway enrichment analyses to illustrate the molecular mechanisms of triptolide in treating DCM.

OB is one of the most important pharmacokinetic parameters [24]. High OB is a major indicator of DL of bioactive molecules and is essential for the molecule to reach target proteins. High DL increases the “hit rate” of drug candidates and is usually used to select the best compounds [25]. In the present study, we found that triptolide had high OB and DL. The Caco-2 and BBB suggested that triptolide had a good permeability in the small-intestinal epithelium and blood-brain barrier. These results indicate that triptolide is a promising drug.

After potential target screening, six DCM-associated targets of triptolide were identified (STAT3, VEGFA, FOS, TNF, TP53, and TGFB1), suggesting that these targets may play key roles in the treatment of DCM by triptolide. STAT3 has been shown to drive the development of DCM, while blockage of STAT3 and its upstream factor, epidermal growth factor receptor, attenuates DCM [26, 27]. VEGFA is involved in the treatment of DCM by SGLT-2 inhibitors [28]. SIRT3-mediated inhibition of FOS ameliorated cardiac fibrosis and inflammation in DCM [29]. TNF-*α* and TGF-*β* are important proinflammatory factor and fibrotic factor, respectively [30–32]. Although the potential role of TP53 in DCM has not been identified, the involvement of TP53 in dilated cardiomyopathy has been reported [33].

To elucidate the functional relationship of these common targets in DCM, we constructed a gene-gene interaction network using the GeneMANIA database. The results revealed that these targets were correlated in terms of coexpression, implying intensive interactions among them. Moreover, these targets also interact intensively with other genes, such as *WWOX*, *SPARC*, *TNFRSF1B*, *JUNB*, and *MDM2*. It has been shown that STAT3 binds to the promoter of *JUNB* to induce inflammation and promote the progression of autoimmune diseases [34, 35]. Anti-VEGF treatment accompanied by inhibition of JUNB has been reported to reduce skin inflammation [36]. SPARC regulated VEGFA signal transduction through the primary angiogenic VEGF receptor [37]. Proinflammatory cytokines (e.g., TNF-*α*) and profibrotic factors (e.g., TGF-*β*1) suppressed the synthesis of SPARC [38]. FOS and JUNB induced by mu-opioid receptor activation formed a functional AP-1 complex and induced the expression of other proteins [39]. The expression of SPARC is markedly increased in the experimental models of cardiac hypertrophy and fibrosis. TNFRSF1B is a TNF-*α* receptor related to inflammatory diseases, such as rheumatoid arthritis [40]. WWOX regulates the expression of TP53 to trigger breast carcinogenesis [41].

The GO analysis revealed that the target genes were enriched in positive regulation of pri-miRNA transcription by RNA polymerase II, regulation of pri-miRNA transcription by RNA polymerase II, and pri-miRNA transcription by RNA polymerase II, indicating that transcriptional regulation is related to the functions of these targets. In addition, the RNA polymerase II transcription factor complex and the nuclear transcription factor complex were identified as the major cell components involved in these biological processes. The major molecular functions of these target genes were core promoter sequence-specific DNA binding, RNA polymerase II transcription factor binding, cytokine receptor binding, and cytokine activity, suggesting that transcriptional regulation of cytokine mRNA expression is highly involved in the treatment of DCM by triptolide.

The KEGG pathway analysis showed that triptolide regulated the activation of pancreatic cancer, rheumatoid arthritis, and AGE-RAGE signaling pathway in diabetic complications in the treatment of DCM. Systemic and local chronic inflammation may increase the risk of pancreatic cancer. Pancreatic cancer-associated inflammatory infiltrate in the tumor microenvironment promotes tumor growth and metastasis [42]. The continuously evolving cross-talk between inflammatory and cancer cells might be direct and contact-dependent. TNF*α*, TGF-*β*, and STAT3 synergistically increased the risk of developing cancers and promoted tumor growth and cancer-associated cachexia [43, 44]. Therefore, these core targets are associated with both DMC and pancreatic cancer. Furthermore, like rheumatoid arthritis, diabetes is an inflammatory disease [45], and the upregulation of inflammatory cytokines has been reported in various mouse models of type 1 or type 2 diabetes-induced DCM, suggesting that inflammation is an important contributor to the development of DCM [46, 47]. Intramyocardial inflammation in diabetic cardiomyopathy has also been reported, as shown by increased expression of inflammatory cytokines (TNF-*α* and TGF-*β*1) [48, 49]. AGEs are predominantly long-lived proteins that become glycated after exposure to sugars, which alters their functional properties [50]. AGEs act via the AGE receptors (RAGE), which are upregulated in diabetic hearts by oxidative stress [51]. The upregulation of AGE and the activation of RAGE result in the activation of the nuclear factor *κ*B signaling, leading to increased expression of the *β*-myosin heavy chain isoform in diabetic hearts. Dehydroepiandrosterone counteracts oxidative stress-induced activation of RAGE in rat models of DCM and normalizes the nuclear factor *κ*B signaling and the upregulation of *β*-myosin heavy chain isoform, thereby contributing to the development of diabetic cardiomyopathy.

## 5. Conclusions

In the present study, six core potential targets (STAT3, VEGFA, FOS, TNF, TP53, and TGFB1) of triptolide in the treatment of DCM are identified by network pharmacology. The GO and KEGG pathway enrichment analyses reveal the mechanism of action of triptolide, that is, triptolide ameliorates DCM through targets related to the inhibition of angiogenesis, synovial hyperplasia, and bone destruction. Our study provides a theoretical basis for further investigation of the therapeutic potential of triptolide for DCM and the use of network pharmacology in drug discovery for other inflammatory diseases. To verify the effects of triptolide and the molecular target genes of anti-DCM, further experimental studies need to be performed in the future. In addition, clinical trials should be conducted to identify the effectiveness of triptolide in humans.

## Figures and Tables

**Table 1:** Pharmacological and molecular properties of triptolide.

Name	MW	AlogP	Hdon	Hacc	OB (%)	Caco-2	BBB	DL	FASA-	TPSA	RBN	HL
Triptolide	360.44	0.87	1	6	51.29	0.25	-0.19	0.68	0.28	84.12	1	4.14

Abbreviations: caco-2: caco-2 permeability; OB: oral bioavailability; Dl: drug likeness; BBB: blood–brain barrier.

**Table 2:** Target genes of triptolide related with CVD.

Num.	Gene ID	Gene symbol	Gene name
1	6774	STAT3	Signal transducer and activator of transcription 3
2	7422	VEGFA	Vascular endothelial growth factor A
3	2353	FOS	Proto-oncogene c-Fos
4	7124	TNF	Tumor necrosis factor
5	7157	TP53	Cellular tumor antigen p53
6	7040	TGFB1	Transforming growth factor beta-1

## Data Availability

All data are available in the manuscript and they are showed in figures, tables and supplement file.

## Supplementary Materials

Supplementary 1 Supplemental Table 1: the predicted targets of triptolide in the TCMSP database.

Supplementary 2 Supplemental Table 2: the common targets of DCM in the CTD database and GeneCards database.
