# Supplementary material for: Potential Mechanisms of Triptolide against Diabetic Cardiomyopathy Based on Network Pharmacology Analysis and Molecular Docking
Source: J Diabetes Res. 2021 Dec 7;2021:9944589. doi: 10.1155/2021/9944589 (PMC8672107; doi:10.1155/2021/9944589)
Supplement: Supplementary 1 — Supplemental Table 1: the predicted targets of triptolide in the TCMSP database. [file 9944589.f1.pdf]

Protein	ID
Transcription factor p65	RELA
Signal transducer and activator of transcription 3	STAT3
Vascular endothelial growth factor A	VEGFA
Apoptosis regulator Bcl-2	BCL2
Proto-oncogene c-Fos	FOS
Cyclin-dependent kinase inhibitor 1	CDKN1C
Urokinase-type plasminogen activator	PLAU
Tumor necrosis factor	TNF
Transcription factor AP-1	JUN
Caspase-3	CASP3
Cellular tumor antigen p53	TP53
Mitogen-activated protein kinase 8	MAPK8
Prostaglandin G/H synthase 2	PTGS2
Signal transducer and activator of transcription 1-alpha/beta	STAT1
Interleukin-8	IL8
Induced myeloid leukemia cell differentiation protein Mcl-1	MCL1
Transforming growth factor beta-1	TGFB1
Interleukin-2	IL2
Interferon gamma	IFNG
Interleukin-4	IL4
Baculoviral IAP repeat-containing protein 4	BIRC4
Beta-defensin 2	BEFB2
T-lymphocyte activation antigen CD80	CD80
T-lymphocyte activation antigen CD86	CD86
C-X-C chemokine receptor type 4	CXCR4
Baculoviral IAP repeat-containing protein 3	BIRC3
Programmed cell death 1 ligand 1	PDCD1L1
Interleukin-23 subunit alpha	IL23A
C-C chemokine receptor type 7	CCR7
T-cell surface glycoprotein CD1a	CD1A
