# Supplementary material for: Potential Mechanisms of Triptolide against Diabetic Cardiomyopathy Based on Network Pharmacology Analysis and Molecular Docking
Source: J Diabetes Res. 2021 Dec 7;2021:9944589. doi: 10.1155/2021/9944589 (PMC8672107; doi:10.1155/2021/9944589)
Supplement: Supplementary 2 — Supplemental Table 2: the common targets of DCM in the CTD database and GeneCards database. [file 9944589.f2.pdf]

The common targets related to DCM were identified in the CTD and GeneCards database

TGFB1
SOD2
AGT
IL6
PPARG
VEGFA
TNF
ICAM1
NOS3
INSR
AKT1
MYH7
IL1B
FOS
IL10
CCL2
CAT
FN1
STAT3
NPPA
INS
SLC2A2
CPT1A
SDHA
EDN1
IGF1
ADIPOQ
NPPB
SLC2A4
TP53
AGTR1
ACE
CRP
